# Therapeutic Mechanism and Key Alkaloids of *Uncaria rhynchophylla* in Alzheimer’s Disease From the Perspective of Pathophysiological Processes

**DOI:** 10.3389/fphar.2021.806984

**Published:** 2021-12-15

**Authors:** Peng Zeng, Hong-Fei Su, Chao-Yuan Ye, Shuo-Wen Qiu, Qing Tian

**Affiliations:** Key Laboratory of Neurological Disease of National Education Ministry, Department of Pathology and Pathophysiology, School of Basic Medicine, Tongji Medical College, Huazhong University of Science and Technology, Wuhan, China

**Keywords:** Alzheimer’s disease, *Uncaria rhynchophylla*, pathophysiological processes, Aβ pathology, tau pathology

## Abstract

Presently, there is a lack of effective disease-modifying drugs for the treatment of Alzheimer’s disease (AD). *Uncaria rhynchophylla* (UR) and its predominant active phytochemicals alkaloids have been studied to treat AD. This study used a novel network pharmacology strategy to identify UR alkaloids against AD from the perspective of AD pathophysiological processes and identified the key alkaloids for specific pathological process. The analysis identified 10 alkaloids from UR based on high-performance liquid chromatography (HPLC) that corresponded to 127 targets correlated with amyloid-β (Aβ) pathology, tau pathology and Alzheimer disease pathway. Based on the number of targets correlated with AD pathophysiological processes, angustoline, angustidine, corynoxine and isocorynoxeine are highly likely to become key phytochemicals in AD treatment. Among the 127 targets, JUN, STAT3, MAPK3, CCND1, MMP2, MAPK8, GSK3B, JAK3, LCK, CCR5, CDK5 and GRIN2B were identified as core targets. Based on the pathological process of AD, angustoline, angustidine and isocorynoxeine were identified as the key UR alkaloids regulating Aβ production and corynoxine, isocorynoxeine, dihydrocorynatheine, isorhynchophylline and hirsutine were identified as key alkaloids that regulate tau phosphorylation. The findings of this study contribute to a more comprehensive understanding of the key alkaloids and mechanisms of UR in the treatment of AD, as well as provide candidate compounds for drug research and development for specific AD pathological processes.

## Introduction

Alzheimer’s disease (AD) is the most common type of dementia characterized by extracellular amyloid-β (Aβ) plaques, intracellular aberrant hyperphosphorylated tau protein, neuronal synaptic dysfunction, and neuronal loss (Wallace and Dalton, 2011; Scheltens et al., 2016; Asaad and Lee, 2018). Clinically, increased concentrations of Aβ42, total-tau and p-tau (p-tau181, p-tau217) in cerebrospinal fluid support the diagnosis of AD dementia (Feng et al., 2021). Moreover, neurofibrillary tangle (formed by tau protein aggregates) topographic distribution in the brain is the basis for Braak neurofibrillary tangle pathological staging of AD (Braak and Braak, 1991). Nowadays, AD has emerged as an important public health issue globally and is characterized by high prevalence, morbidity and mortality, and high burden of economic. Currently, around 9.8 million people in China and 47 million people worldwide suffer from dementia, which is expected to more than triple by 2050 (about 131 million) (Tiwari et al., 2019). One of the major challenges in AD is no effective therapeutic strategy for AD. There are five drugs currently used clinically to improve the symptoms of AD, including cholinesterase inhibitors and the N-methyl-D-aspartic acid (NMDA) receptor antagonist (Melnikova, 2007). These drugs are symptomatic treatments and are highly patient dependent. Moreover, these drugs have undesirable side effects in AD patients, including hypertensive crisis, nausea, diarrhea, and vomiting (Inglis, 2002). It is worth noting that the Food and Drug Administration recently approved Aduhelm (aducanumab), an antibody that targets Aβ, for the treatment of AD (Tanzi, 2021). As the first approved drug with a putative disease-modifying mechanism for AD treatment, it has been met with a fair degree of skepticism and controversy (Lalli et al., 2021; Rabinovici, 2021). Since 2003, no other presumed AD modification drugs or new symptomatic treatments have been approved (Cummings, 2021). Therefore, it is extremely urgent to find drugs that can prevent or delay the onset or progression of AD.

Chinese herbal medicine has been widely used to treat cognitive impairment or dementia (Liu L. et al., 2016; Zeng et al., 2021a). *Uncaria rhynchophylla* (UR), named Gouteng in Chinese, is a traditional Chinese herb native to China, Japan and Vietnam that has been used for more than a thousand years. It is utilized to extinguish the wind, arrest convulsions, clear heat, and pacify the liver. Clinically, UR is widely used to treat dizziness, headaches, epilepsy, and hypertension (Hsieh et al., 1999a; Hsieh et al., 1999b; Zhou and Zhou, 2010; Liu L. et al., 2016). These effects are largely attributed to alkaloids, which is the predominant active phytochemicals in UR and comprise about 0.2% (Zhou and Zhou, 2010). Of these UR alkaloids, the contents of rhynchophylline (RHY) and isorhynchophylline (IRN) were the highest, accounting for 28–50% and 15%, respectively (Shi et al., 2003). In 5xFAD mice, a transgenic AD model with rapid Aβ plaque deposition, oral administration UR extract (400 mg/kg/day for 4 weeks) significantly alleviates Aβ deposition and Aβ-mediated neuropathology (Shin et al., 2018). RHY and IRN extracted from UR have protective effects on Aβ-induced neuronal toxicity, and its mechanism involves intracellular calcium overloading and tau protein hyperphosphorylation (Xian et al., 2012). In PC12 cells, isorhynchophyline increases phosphorylation of Akt and glycogen synthase kinase 3β (GSK3β, encoded by GSK3B) levels against Aβ_25-35_ induced apoptosis (Xian et al., 2013). The aim of this study was to find novel drugs that produce an enduring change in the clinical progression of AD, particularly through a variety of intermediate mechanisms such as the effect on Aβ or tau.

To uncover the therapeutic mechanism of UR alkaloids in AD, a novel network pharmacology strategy from the perspective of AD pathophysiological processes was employed. As a result, we analyzed the targets of associated with correlated with Aβ pathology, tau pathology and Alzheimer disease pathway, and further identified key alkaloids for Aβ production and degradation, and tau phosphorylation. To increase the credibility of the data source, 10 alkaloids extracted from UR were obtained from a recent high-performance liquid chromatography (HPLC) study (Zheng et al., 2021). This avoids that the key phytochemicals are widely distributed non-specific components such as quercetin, β-sitosterol and kaempferol. Furthermore, we also used the human high-throughput omics data to validate the targets of UR against AD. Our study offers new insight into the mechanisms of UR alkaloids and provides a more specific and effective treatment AD.

## Materials and Methods

### Determination of the Main Alkaloids of UR and Pharmacological Parameters Evaluation

A total of 10 main alkaloids of UR were obtained from a recent study based on HPLC (Zheng et al., 2021). HERB database (http://herb.ac.cn/) (Fang et al., 2021) was employed to retrieve the number of herbs that contain above UR alkaloids. Lipinski’s rule of five (RO5), i.e., molecule weight (MW) < 500, number of hydrogen bond donors (Hdon) ≤ 5, number of hydrogen bond acceptors (Hacc) ≤ 10, lipid-water partition coefficient (LogP) ≤ 5 and number of rotatable bonds (Rbon) ≤ 10, has been extensively used to evaluate bioavailability based on the structures of compounds (Lipinski et al., 2001). Here, we employed the SwissADME web tool (www.swissadme.ch) (Daina et al., 2017; Zeng et al., 2021b) to evaluate the compounds according to RO5. The toxicological parameters (hepatotoxicity, carcinogenicity, immunotoxicity, mutagenicity and acute oral toxicity) of the identified UR alkaloids were determined *via* Protox II webserver (https://tox-new.charite.de/protox_II/) (Banerjee et al., 2018). The Encyclopedia of Traditional Chinese Medicine database (ETCM, http://www.tcmip.cn/ETCM/) (Xu et al., 2019) was utilized to retrieve the drug-likeness parameters of the UR alkaloids. The PubMed database (www.ncbi.nlm.nih.gov/pubmed) was used to search the literature containing the content of UR alkaloids and the permeability of blood-brain barrier (BBB).

### Collection of the Targets of UR Alkaloids

Canonical SMILES of the main alkaloids in UR were extracted from PubChem database (https://pubchem.ncbi.nlm.nih.gov/) (Kim et al., 2016). The 2-dimensional chemical structures were generated by ChemDraw Ultra 8.0 software (Cambridge, MA, United States). The targets of the UR alkaloids were obtained using SwissTargetPrediction (http://www.swisstargetprediction.ch/) (Daina et al., 2019). Specifically, canonical SMILES were input into the SwissTargetPrediction, and the target species was set as *Homo sapiens*. Subsequently, target information was collected and organized using Microsoft Excel software (version 2019, Redmond, WA, United States).

### Screening UR Alkaloids Targets Correlated With AD Pathology

PathCards database (https://pathcards.genecards.org/) (Belinky et al., 2015) is an integrated database of human biological pathways and their annotations. We collected 369 genes involved in Alzheimer disease pathway from the PathCards database. Afterward, we took the intersection of Alzheimer disease pathway genes and the potential targets of UR alkaloids and the common targets were the UR targets involved in Alzheimer disease pathway. Additionally, we also used the AlzData (http://www.alzdata.org/) (Xu et al., 2018) to screen the UR targets involved in Aβ and tau pathology (key neuropathological hallmarks of AD pathology) in the “Convergent functional genomic (CFG) Ranks” module. Results were collated and summarised using Microsoft Excel software (version 2019, Redmond, WA, United States). The normalized expression levels of UR targets related to Aβ and tau pathology in the control and AD groups in the Gene Expression Omnibus (GEO) dataset were analyzed with the “Differential expression” module of AlzData. GraphPad Prism software (version 8.0, San Diego, CA, United States) was used for graphical visualization. Values are presented as the mean ± standard deviation (SD).

### Functional Classification of Potential Targets of UR Alkaloids

The functional annotation and the classification of UR targets were performed using the Panther classification system (http://pantherdb.org/) (Mi et al., 2007). The UR targets were uploaded to the Panther classification system the organism was limited to *Homo sapiens*. GraphPad Prism software (version 8.0, San Diego, CA, United States) was used to graph results. Sankey diagrams were plotted using OriginPro 2021 software (OriginLab Corporation, Northampton, MA, United States).

### Protein-Protein Interaction Network Construction and Screened its Core Targets

The PPI network of the target proteins was collected using the latest version of STRING database (version 11.5, https://string-db.org/) (Szklarczyk et al., 2021) and visualized using Cytoscape software (version 3.7.1) (Shannon et al., 2003). The organism was set to *Homo sapiens* and only PPIs with an interaction score exceeding the threshold of 0.4 were included. In the PPI network, degree refers to the number of other nodes directly connected to a node. The higher the degree is, the more important the node is in the PPI network. The degree values were calculated using Network Analysis (a Cytoscape plugin), and the top 10 targets ranked by degree were selected and identified as core targets.

### Gene Ontology and the Kyoto Encyclopedia of Genes and Genomes Pathway Enrichment Analysis

ClusterProfiler R package (version 4.0.2) (Yu et al., 2012) was used for enrichment analysis, including GO enrichment and KEGG pathway enrichment. The *p* values were adjusted using a Benjamini-Hochberg (BH) approach. Statistical significance was denoted if adjust *p* value <0.05. Only GO terms in the category biological process were considered in this study. Top 20 GO biological processes and KEGG pathways sorted by the *p* value were visualized using an online tool (http://www.bioinformatics.com.cn/) (Zeng et al., 2021c). We used the pathview software Microsoft Office Powerpoint (version 2019, Microsoft, Seattle, WA) to superimpose the UR targets involved in the Alzheimer disease pathway (hsa05010) and corresponding alkaloids on KEGG pathway map.

### Molecular Docking Simulations

To validate the binding of core targets to its corresponding UR alkaloids, the 3D molecular structure of alkaloids was downloaded from the PubChem database (https://pubchem.ncbi.nlm.nih.gov/) (Kim et al., 2016). The crystal structure of target proteins was obtained from the RCSB Protein Data Bank (PDB database, http://www.rcsb.org/) (Westbrook et al., 2002). The LeDock program (http://www.lephar.com/software.htm) (Wang et al., 2016) was applied for molecular docking studies. Two-dimensional hydrogen bonding diagrams were generated using LigPlot (https://www.ebi.ac.uk/thornton-srv/software/LIGPLOT/) (Laskowski and Swindells, 2011).

## Results

### Main Alkaloids of UR and Their Pharmacological and Molecular Properties

**FIGURE 1:**
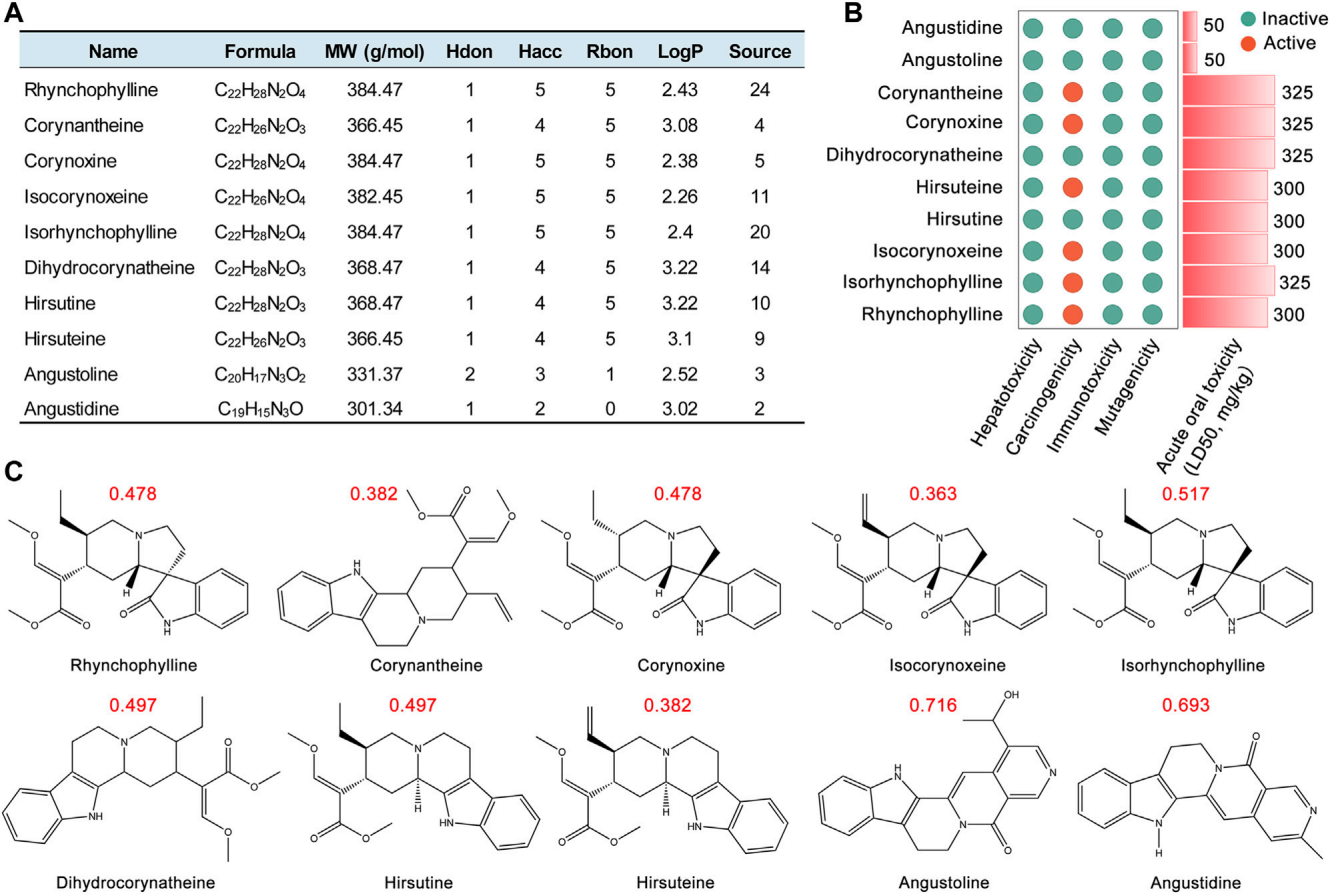
Evaluation of pharmacological and toxicological parameters of main alkaloids from *Uncaria rhynchophylla* (UR). **(A)** Pharmacological and molecular properties of the main alkaloids in UR. **(B)** The toxicological parameters including hepatotoxicity, carcinogenicity, immunotoxicity, mutagenicity and acute oral toxicity of the main alkaloids in UR. **(C)** The chemical structure and drug-likeness parameters of the main alkaloids extracted from UR. Red numbers represent drug-likeness values. MW, molecule weight; Hdon, hydrogen bond donors; Hacc, hydrogen bond acceptors; Rbon, rotatable bonds; LogP, lipid-water partition coefficient.

This study focused on the alkaloids, the major pharmacological active constituents of UR, and obtained 10 main alkaloids from a recent HPLC study (Zheng et al., 2021). RO5 was employed to evaluate the drug-likeness properties and *in vivo* absorption abilities of chemical compounds (Lipinski et al., 2001; Lipinski, 2004). SwissADME prediction (Daina et al., 2017; Zeng et al., 2021b) showed that all the main alkaloids complied with RO5 (Figure 1A). We retrieved HERB database (Fang et al., 2021) to analyze the distribution of these UR alkaloids in traditional Chinese medicines. The results showed that the distribution of UR alkaloids in UR was highly specific. Specifically, angustidine, angustoline and corynantheine only exist in 2, 3 and 4 kinds of traditional Chinese medicines including UR, respectively (Figure 1A). The most widely distributed UR alkaloid RHY is only found in 24 kinds of traditional Chinese medicines. The contents of hirsuteine, hirsutine and RHY were 17.14, 12.86 and 58.21 mg/g of UR total alkaloids, respectively (Zhang et al., 2013). Another study showed that hirsuteine, hirsutine, isocorynoxeine, IRN and RHY were 12.75, 16.25, 13.50, 11.50 and 8.75 mg/g of UR refined alkaloids (Feng et al., 2015). AD is a common neurological disease, studying the permeability of the blood brain barrier of the UR alkaloids may help to better understand its central nervous system activities. *In vivo* experiments, the corresponding alkaloids can be detected in the brain after intraperitoneal injection or intravenous injection of hirsutine, hirsuteine (Imamura et al., 2011; Zhou et al., 2020) and RHY (Lee et al., 2014) (Supplementary Figure S1). In the experiment of BBB permeability *in vitro*, hirsutine, hirsuteine, isocorynoxeine, IRN and RHY could pass through the brain endothelial cells in culture conditions (Imamura et al., 2011; Zhang et al., 2017). These evidences show that UR alkaloids can enter the brain and have the material basis to treat AD.

Drug toxicology is one of the key fields of preclinical research. The protox II webserver (Banerjee et al., 2018) was used to predict toxicological parameters such as hepatotoxicity, carcinogenicity, immunotoxicity, mutagenicity and acute oral toxicity (LD50, mg/kg). None of the UR alkaloids showed hepatotoxicity, immunotoxicity and mutagenicity, while corynantheine, corynoxine, hirsuteine, isocorynoxeine, IRN and RHY have the risk of carcinogenicity (Figure 1B). Moreover, angustidine and angustoline showed the lowest LD50 values (50 mg/kg). The chemical structure and drug-likeness of 10 main alkaloids from UR are shown in Figure 1C. All the UR alkaloids displayed favorable drug-likeness parameters (shown in red font). These results indicate that these 10 UR alkaloids showed good pharmacological parameters and molecular properties, which could be used as candidate drugs for follow-up analysis.

### Screening UR Alkaloids Targets Correlated With Aβ Pathology, Tau Pathology and Alzheimer Disease Pathway

**FIGURE 2:**
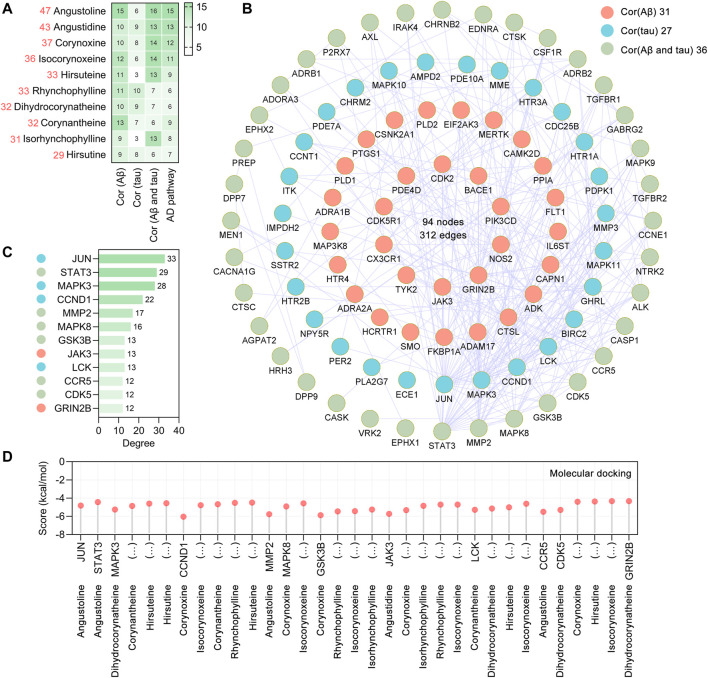
Aβ, tau pathology and Alzheimer disease pathway related UR targets and their PPI network. **(A)** The heatmap shows the number of UR targets correlated with Aβ pathology, tau pathology, Aβ and tau pathology, and Alzheimer disease pathway, respectively. The red numbers on the left represent the total number of targets correlated with Aβ pathology, tau pathology and Alzheimer disease pathway. **(B)** The PPI network was constructed for the 107 potential targets of UR associated with Aβ and tau pathology. Nodes in red, cyan and green represent UR targets correlated with Aβ pathology, tau pathology, Aβ and tau pathology, respectively. **(C)** The top 10 core targets were ranked by degree. The color of the left node of the core targets was correlated with Aβ pathology, tau pathology, Aβ and tau pathology, respectively. **(D)** Molecular docking of UR alkaloids with core targets correlated with Aβ and tau pathology.

By retrieving the SwissTargetPrediction database (Daina et al., 2019), a total of 365 potential targets of UR alkaloids were identified based on their structures. To explore the therapeutic mechanism of the main alkaloids in the treatment of AD, a novel strategy was used to screen the therapeutic targets from the perspective of AD pathophysiological processes in this study. We approached this strategy in two ways. First, the AlzData database (Xu et al., 2018) was used to screen the UR targets involved in Aβ and tau pathology (key neuropathological hallmarks of AD pathology), and a total of 107 targets were obtained. Second, intersection of UR alkaloids targets and 369 Alzheimer disease pathway targets was taken, and a total of 36 common targets were screened out. After all the extracted UR targets were pooled, a total of 127 UR alkaloids targets were obtained. Detailed information of the 127 targets is provided in Supplementary Table S1. The number of UR targets correlated with Aβ pathology, tau pathology and Alzheimer disease pathway is shown in Figure 2A. Specifically, angustoline has the largest number of targets (47 targets) correlated with pathophysiological processes, followed by angustidine (43 targets), corynoxine (37 targets) and isocorynoxeine (36 targets), indicating that these alkaloids are highly likely to become key phytochemicals in AD treatment. To identify the core targets associated with AD pathology, the PPI network was constructed using the STRING database (version 11.5) (Szklarczyk et al., 2021) and visualized in Cytoscape (version 3.7.1). The targets in the Alzheimer disease pathway are not all related to Aβ and tau pathology. There are 16 UR alkaloid targets not only related to Aβ pathology and tau pathology, but also involved in the Alzheimer disease pathway. Therefore, we constructed a PPI network from 107 targets related to Aβ and tau pathology and a PPI network with a total of 94 nodes and 312 edges and an average node degree of 6.64 was generated (Figure 2B). JUN, STAT3, MAPK3, CCND1, MMP2, MAPK8, GSK3B, JAK3, LCK, CCR5, CDK5 and GRIN2B, which are ranked by degree, were identified as core targets (Figure 2C). Among these core targets, JUN showed the highest degree (33), followed by STAT3 (degree = 29) and MAPK3 (degree = 28). We further analyzed the number of core targets and corresponding core targets of UR alkaloids. Isocorynoxeine has the largest number of core targets including CCND1, MAPK8, GSK3B, JAK3, LCK and CDK5, followed by corynoxine (5 targets, CCND1, MAPK8, GSK3B, JAK3 and CDK5), dihydrocorynatheine (4 targets, MAPK3, LCK, CDK5 AND GRIN2B), angustoline (3 targets), corynantheine (3 targets), hirsuteine (3 targets) and RHY (3 targets) (Supplementary Figure S2).

Molecular docking analysis was applied to validate the binding of core targets and its corresponding UR alkaloids, and the lowest energy docking model was selected. The results showed that all the core targets were well combined with the corresponding UR alkaloids (Figure 2D). Among these targets, angustidine showed the highest binding energy with STAT3 and JAK3, with score values of −4.44 and −5.72 kcal/mol, respectively; angustoline showed the highest binding energy with JUN and MMP2, with score values of −4.82 and −5.76 kcal/mol, respectively; corynoxine showed the highest binding energy with CCND1, MAPK8, GSK3B, JAK3 and CDK5, with score values of −6.04, −4.91, −5.87, −5.32 and −4.4 kcal/mol, respectively. The above results demonstrated that these UR alkaloids, which are closely related to the pathology of AD, may play a critical role in the treatment of AD.

### GEO Dataset Analysis of UR Targets Related to Aβ and Tau Pathology

**FIGURE 3:**
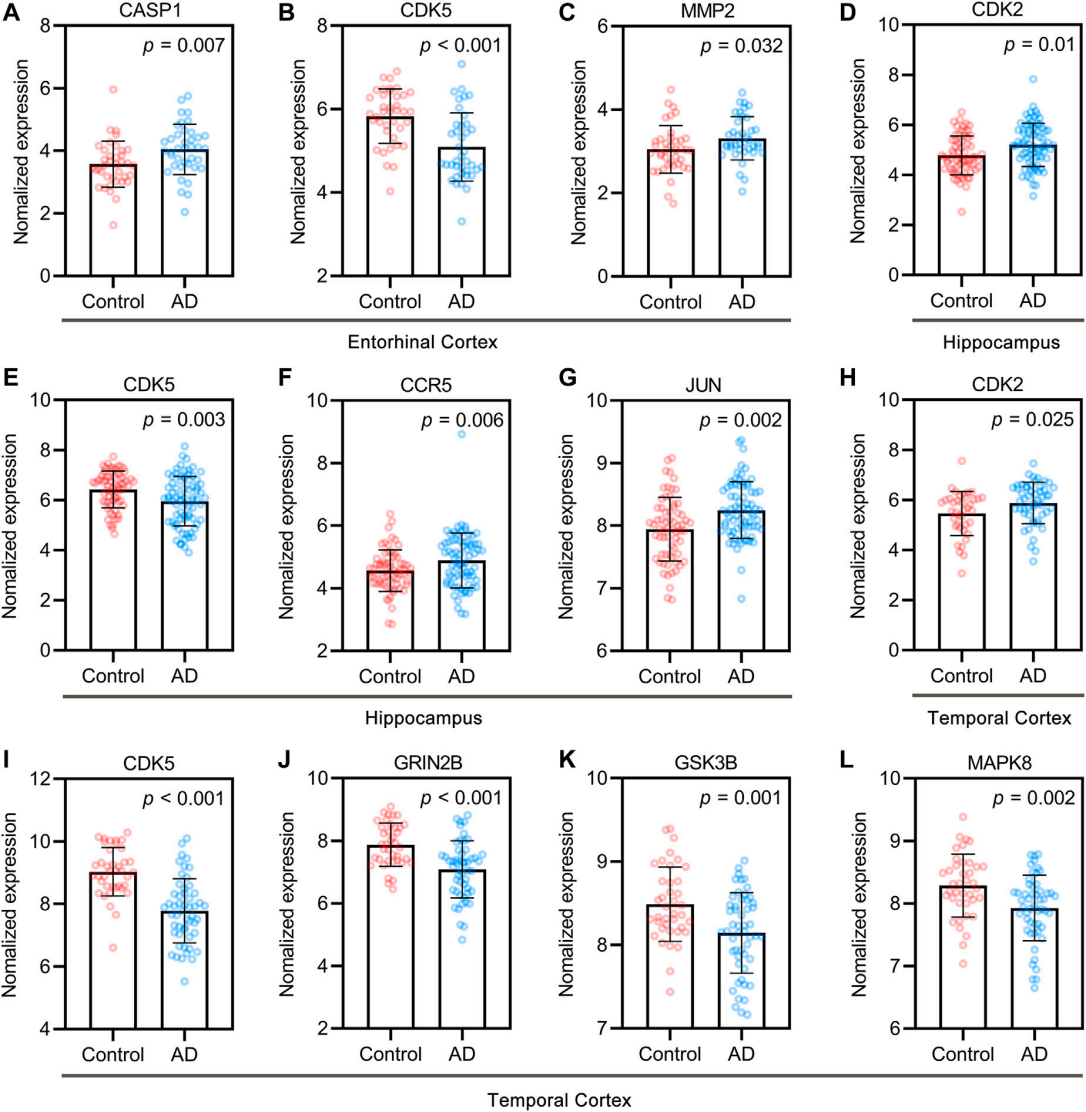
Core targets of UR alkaloids in the control and AD groups of the GEO dataset. Differentially expressed targets in entorhinal cortex **(A–C)**, hippocampus **(D–G)**, and temporal cortex **(H–L)**. Entorhinal cortex, n = 39 in each group. Hippocampus, n = 66 in the healthy control group, n = 74 in the AD group. Temporal cortex, n = 39 in the healthy control group, n = 52 in the AD group. Values are presented as the mean ± standard deviation (SD).

Based on the “Differential expression” module of the AlzData database, we analyzed the normalized expression values of UR targets related to Aβ and tau pathology in the healthy control and AD groups in the GEO (Figure 3). Of the targets, CASP1, CDK5 and MMP2 were differentially expressed in the entorhinal cortex (Figures 3A–C), CDK2, CDK5, CCR5 and JUN were differentially expressed in the hippocampus (Figures 3D–G), and CDK2, CDK5, GRIN2B, GSK3B, MAPK8 were differentially expressed in the temporal cortex (Figures 3H–L). Of note, increased CDK5 and GSK3B activity are closely related to tau hyperphosphorylation and NFT formation. Above results pointed out that these UR targets associated with Aβ and tau pathology play an important role in AD brain.

### Functional Classification of UR Targets Correlated With Aβ Pathology, Tau Pathology and Alzheimer Disease Pathway

**FIGURE 4:**
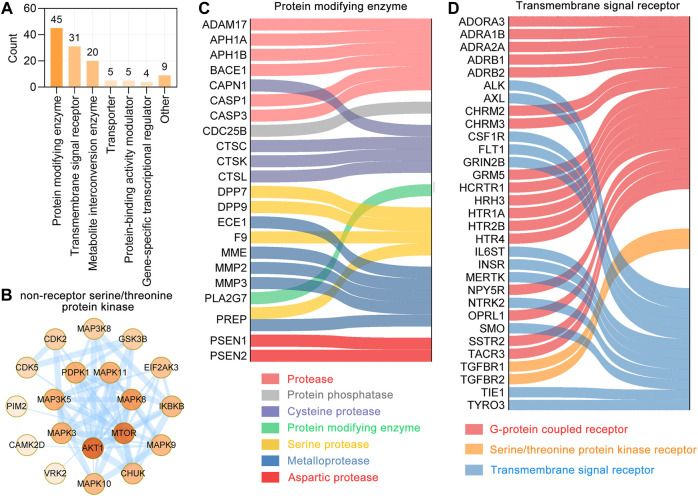
Functional classification of UR targets which correlated with Aβ, tau pathology and Alzheimer disease pathway. **(A)** Panther classification categorized target proteins of UR. The numbers above each bar chart represent the number of the targets in the given functional class. **(B)** The PPI network of targets involved in the non-receptor serine/threonine protein kinase. Colors of the nodes represent the degree, the darker the color the greater the degree. The thickness of the edges represents the combined score. **(C,D)** Sankey diagram of UR targets involved in protein modifying enzyme (PC00260) and transmembrane signal receptor (PC00197). The color of the sankey diagram represents its corresponding functional classification.

We performed a functional classification using Panther classification system (Mi et al., 2007), from which 119 out 127 UR targets could be functionally classified. The 119 UR targets correlated with Aβ pathology, tau pathology and Alzheimer disease pathway were categorized into 7 different classes based on their cellular function, of which protein modifying enzyme (PC00260, 45 targets) was the most enriched class, followed by transmembrane signal receptor (PC00197, 31 targets) and metabolite interconversion enzyme (PC00262, 20 targets) (Figure 4A). Protein kinases are phosphorylase that regulate specific amino acids and are essential for the conversion of active forms of proteins. In protein modifying enzymes, 21 targets are non-receptor serine/threonine protein kinase and form a complex PPI network with 20 nodes and 56 edges (Figure 4B). Among these kinases, CDK5 and GSK3B are key kinases regulating tau phosphorylation. For other protein modifying enzymes, ECE1, MME, MMP2, MMP3, PREP belong to metalloproteases; ADAM17, APH1A, APH1B, BACE1, CASP1 and CASP3 belongs to protease; and CAPN1, CTSC, CTSK and CTSL are cysteine proteases (Figure 4C). Transmembrane signal receptors are cell surface, membrane-anchored proteins that bind to external ligand molecules. For transmembrane signal receptors, 17 UR potential targets were G-protein coupled receptor, followed by 12 UR targets were transmembrane signal receptor (Figure 4D). The above findings indicate that the therapeutic targets of UR alkaloids for AD are involved in multiple critical biological functions.

### Potential Synergistic Mechanisms of UR Alkaloids Against AD

#### GO Biological Process Enrichment Analysis

**FIGURE 5:**
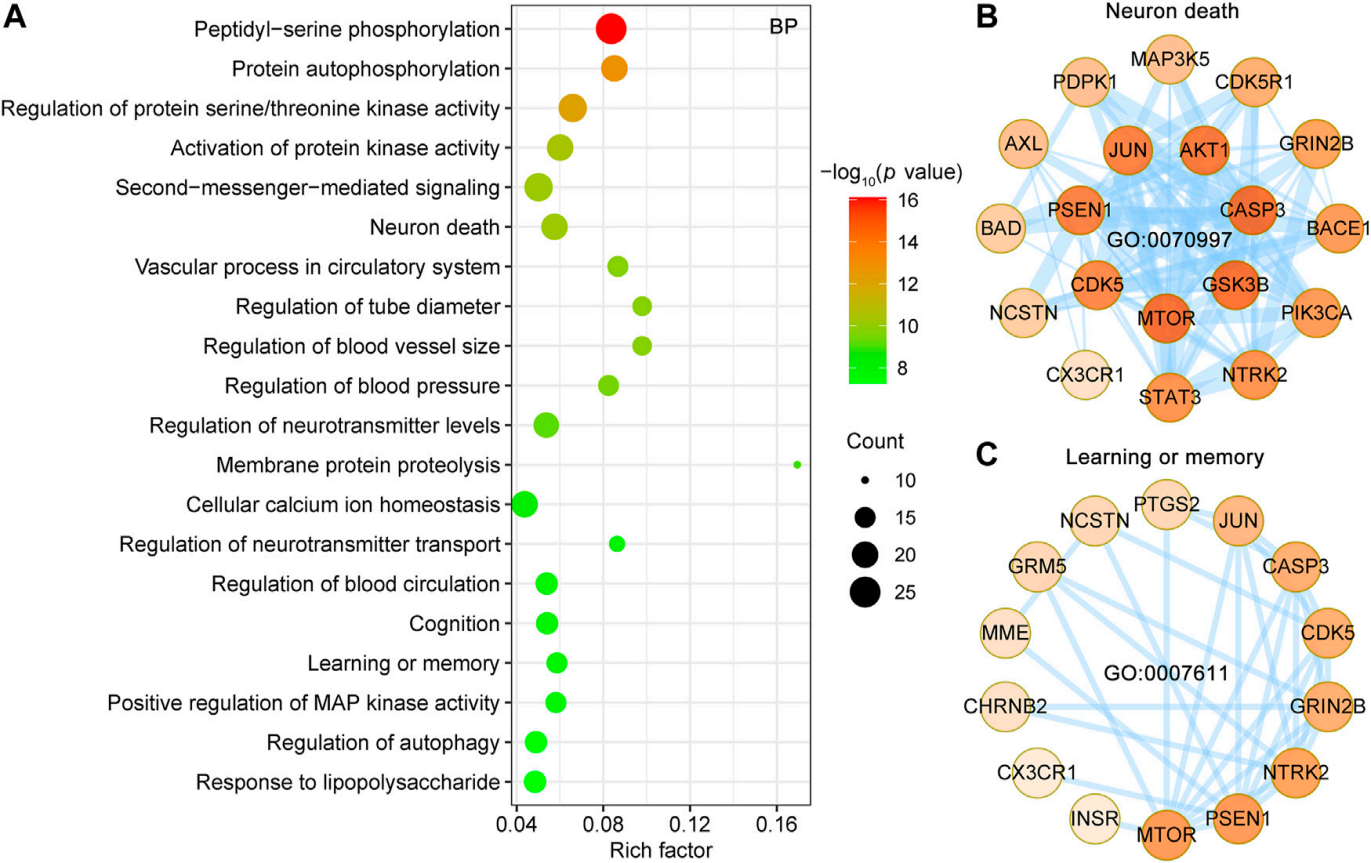
Enrichment analysis of GO biological process of UR targets associated with Aβ, tau pathology and Alzheimer disease pathway. **(A)** Top 20 bubble chart of biological process (BP) of GO enrichment analysis. The X-axis represents the rich factor, bubble size represents the count of targets enriched in terms and the color represents the *p* value. **(B,C)** PPI network of UR targets involved in neuron death (GO:0070997) and learning or memory (GO:0007611). The darker the color, the higher the degree. The thickness of the edges represents the combined score.

To further analyze synergistic mechanisms of UR alkaloid targets for AD, the clusterProfiler R package (Yu et al., 2012) was employed to analyze the 127 UR targets associated with AD pathological processes in GO biological process and KEGG pathway. Top 20 enriched GO BP terms ranked by the adjust *p* value are shown (Figure 5A). The primary enriched BP terms were peptidyl-serine phosphorylation (GO:0018105), protein autophosphorylation (GO:0046777), regulation of protein serine/threonine kinase activity (GO:0071902), activation of protein kinase activity (GO:0032147), second-messenger-mediated signaling (GO:0019932), neuron death (GO:0070997) and so on. Notably, peptidyl-serine phosphorylation exhibited the greatest number of target connections (degree = 25), followed by regulation of protein serine/threonine kinase activity and second-messenger-mediated signaling (degree = 22). Twenty potential UR targets involved in neuron death (GO:0070997) formed a complex PPI network, which included 19 nodes and 84 edges (Figure 5B). In particular, 5 out of 20 UR targets involved in neuron death were core targets (CDK5, GRIN2B, GSK3B, JUN and STAT3). Progressive impairment of memory is one of the hallmarks of AD, and 15 UR targets are involved in learning or memory (GO:0007611) and form a PPI network with 14 nodes and 34 edges (Figure 5C). Among these targets, CDK5, GRIN2B, JUN were core targets. These findings suggest that these UR targets related to AD pathological processes are involved in a variety of biological processes.

### KEGG Pathway Enrichment Analyses for Targets of UR Alkaloids Against AD

**FIGURE 6:**
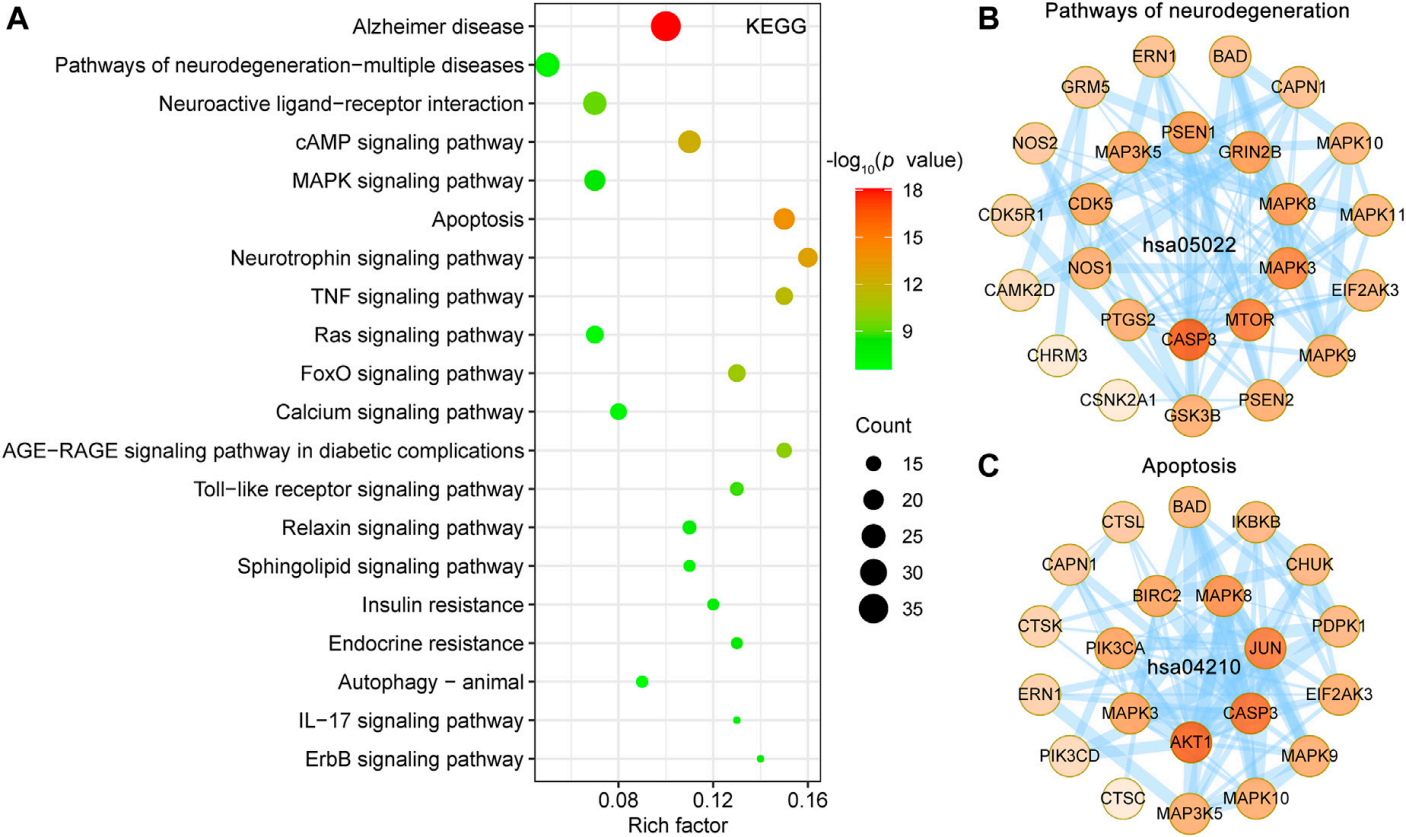
KEGG pathway enrichment analysis of UR targets associated with Aβ, tau pathology and Alzheimer disease pathway. **(A)** The top 20 KEGG pathways are presented in the bubble chart. X-axis, rich factor; bubble size, the number of targets enriched; bubble color, *p* value. **(B,C)** PPI network construction for targets involved in pathways of neurodegeneration-multiple diseases (hsa05022) and apoptosis (hsa04210). The darker the color, the higher the degree. The thickness of the edges represents the combined score.

KEGG pathway database is a collection of manually drawn pathway maps of molecular interactions. KEGG pathways mainly involved Alzheimer disease (hsa05010), pathways of neurodegeneration—multiple diseases (hsa05022), neuroactive ligand-receptor interaction (hsa04080), cAMP signaling pathway (hsa04024), apoptosis (hsa04210) and MAPK signaling pathway (hsa04010) (Figure 6A). Detailed information on the KEGG pathway enrichment analysis is shown in Supplementary Table S2. The Alzheimer disease pathway (hsa05010, *p* = 7.47E-19, 36 targets) is the most significantly enriched pathway, belongs to the Human Diseases category pathway, and is mainly associated with the production and clearance of Aβ and aberrant tau hyperphosphorylation. A PPI network of the 25 UR targets involved in pathways of neurodegeneration-multiple diseases (hsa05022) in Figure 6B and contains 25 nodes and 96 edges. Among these targets, CDK5, GRIN2B, GSK3B, MAPK3 and MAPK8 were core targets. Furthermore, apoptosis (hsa04210), a dominant pathway of neuronal destruction in AD, was also significantly enriched (Figure 6C). The UR targets involved in the apoptosis pathway (hsa04210) form a PPI network with 21 nodes and 84 edges, in which ATK1, CASP3 and JUN play an important role. These results implicated the involvement of multiple targets and various pathways in multiple synergistic effects of UR alkaloids against AD.

### Analysis of Key Alkaloids of UR Against AD Based on Alzheimer Disease Pathway

**FIGURE 7:**
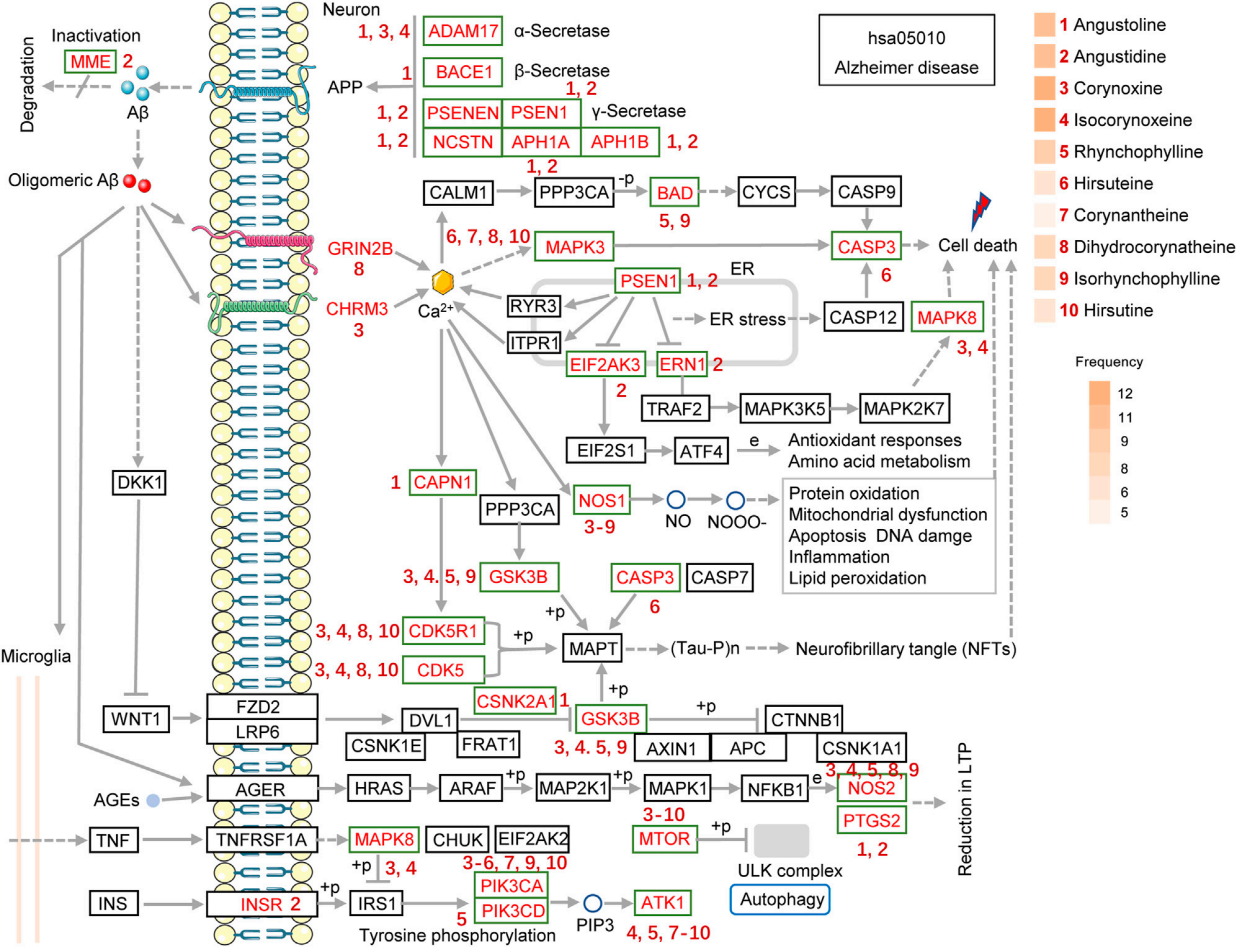
The UR targets involved in the Alzheimer disease pathway (hsa05010) are presented in the mechanistic diagram of AD pathology. The red font targets represent the UR targets involved in the Alzheimer disease pathway. The brownish red numbers 1 to 10 represent the main alkaloids of UR. The gradient color on the left side of UR alkaloids represents its frequencies. The number next to the target indicates the UR alkaloid that can act on the corresponding target.

To identify key alkaloids of UR against AD, pathview software was used to visualize the UR targets involved in the Alzheimer disease pathway and corresponding alkaloids (Figure 7). The red font targets represent the UR targets involved in the Alzheimer disease pathway. The brownish red numbers 1 to 10 represent the main alkaloids of UR. Senile plaques deposition is a result of imbalanced Aβ production and degradation. Angustidine could act on MME and participate in the degradation of Aβ. The generation of Aβ depends on the cleavage of APP by α-secretase (ADAM10 and ADAM17), β-secretase (BACE1and BACE2) and γ-secretase (APH1A, APH1B, NCSTN, PSEN1, PSEN2 and PSENEN). Judging from Figure 7, angustoline and angustidine are the key alkaloids that regulate the production of Aβ. As another pathological process of AD, tau protein phosphorylation is mainly regulated by CDK5, CDK5R1, CASP3 and GSK3B. Given this, corynoxine, isocorynoxeine, dihydrocorynatheine, IRN and hirsutine are identified as key alkaloids that regulate tau phosphorylation. Among the 4 alkaloids that interact with GSK3B, corynoxine has the highest similarity to the selective GSK3β inhibitor AR-A014418 (Supplementary Figure S3). Specifically, AR-A014418 and corynoxine formed potential interactions with residues Arg220 and Ser66 of GSK3β through hydrogen bonds. This suggests that corynoxine is more likely to be a potential GSK3β inhibitor.

It should be noted that dihydrocorynatheine interacts with GRIN2B, which forms NMDA receptor, and corynoxine interacts with CHRM3, which forms G protein-coupled receptor, thus affecting downstream calcium signaling pathway and cell death. It is well known that oligomeric forms of Aβ is an important inflammatory stimulus of neuronal cells. Angustoline and angustidine can interact with PTGS2, and corynoxine, isocorynoxeine, RHY, dihydrocorynatheine, IRN can interact with NOS2 to improve the inflammation induced by oligomeric Aβ. We further counted the number of times each UR alkaloids appeared in the Alzheimer disease pathway, and plotted its frequencies. Corynoxine and isocorynoxeine have the highest frequency (12 times), followed by angustoline (11 times), angustidine (11 times), RHY (9 times), dihydrocorynatheine (8 times) and IRN (8 times). The results above strongly suggest that a variety of UR alkaloids act on different important pathological processes of AD and play a key role in the treatment of AD.

## Discussion

Here, key phytochemicals and potential mechanisms of UR alkaloids in the treatment of AD were analyzed from the perspective of AD pathophysiological processes. This study has several advantages over traditional network pharmacology approaches. First, this study adopted a novel strategy to identify UR alkaloids against AD from the perspective of AD pathophysiological processes and identified the key alkaloids for specific pathological process. This avoids the subjectivity of potential target selection for the disease and improves the accuracy of UR targets screening against AD. Second, the present study focused on alkaloids, the bioactive constituents of UR, and obtained 10 main alkaloids from a recent HPLC study (Zheng et al., 2021). This prevents the use of public databases to screen out a large number of non-specific components. The alkaloid source analysis also proved that the distribution of these alkaloids in UR was highly specific. Third, the pharmacological and molecular properties of UR alkaloids were systematically evaluated. Except for RO5, we also evaluated the content, toxicological parameters, and drug-likeness properties of UR alkaloids, and reviewed the literature for alkaloids that can cross BBB. It affords a comprehensive insight into these UR alkaloids. Finally, we also used the human high-throughput omics data to validate the targets of UR related to Aβ and tau pathology in AD brain. Of course, there were some limitations in this study. First, the complex dose-effect relationship and toxicological evaluation *in vivo* were not considered. Second, the current study is still static network analysis, which contradicts the dynamic changes of disease occurrence and development. Finally, chemical changes that occur during various drug manufacturing processes are not considered in this study.

The accumulation of Aβ peptide, the main component of amyloid plaques, is a key and initiating factor in the pathogenesis of AD. The homeostatic balance between Aβ production and clearance is critical to maintain brain health. Significant Aβ deposition was also observed in the cerebral and leptomeningeal vessels of AD patients (Jellinger et al., 2007). Aβ is derived from the APP, which generates various polypeptides *via* sequential cleaving by α-secretase, β-secretase, and γ-secretase. It is noteworthy that APP is widely expressed in many tissues except the brain, including the adrenal gland, kidney, heart, liver, spleen, pancreas, muscles, and various blood and endothelial cell (Yankner and Mesulam, 1991; Roher et al., 2009). Peripheral derived Aβ is one of the many sources of total Aβ, and Aβ levels in peripheral tissues and brain were lower in the cognitively normal elderly than in AD patients (Roher et al., 2009). Compared with healthy controls, Aβ clearance *via* the BBB is reduced by about 30% in AD patients (Mawuenyega et al., 2010). This might suggest that active clearance of excess peripheral Aβ is also a promising strategy for AD therapy. In transgenic AD mouse model (5xFAD), UR ethanol extract (including RHY) significantly reduced Aβ aggregation and ameliorated AD-related pathologies (neuronal loss, synaptic degeneration, neuroinflammation, and neurogenesis) (Shin et al., 2018). In this study, angustoline, angustidine and isocorynoxeine were identified as the key UR alkaloids regulating Aβ production. Among these alkaloids, angustoline and angustidine have attracted much more of our attention. Angustoline and angustidine can act on three kinds of secretases including α-secretase, β-secretase, and γ-secretase. MME is considered the most important Aβ-degrading enzymes in the prevention of AD pathology (Shin et al., 2018; Miners et al., 2012). Angustidine also act on MME and participate in the degradation of Aβ. By retrieving the literature, we found that 5 UR alkaloids (hirsutine, hirsuteine, isocorynoxeine, IRN and RHY) have been reported to cross the BBB into the brain (Imamura et al., 2011; Lee et al., 2014; Zhang et al., 2017; Zhou et al., 2020). This suggests that UR alkaloids treat AD in a central and peripheral manner. CCR5, which significantly positively associated with both Aβ and tau pathology, is a kind of cytokine belonging to the β chemokine receptor family of integral membrane proteins (Power et al., 1995). In the brain, CCR5 is highly expressed in microglia and to a lesser extent in astrocytes, and neurons (Fantuzzi et al., 2019). In AD patients and AD rodent models, increased expression of CCR5 suggests a correlation between AD and CCR5 expression (Necula et al., 2021). CCR5 expression is strongly related to microglia and inflammation, which accelerate the development of AD (Li and Zhu, 2019). The core target CCR5 is a potential target of angustoline and should be considered in future studies.

GSK3β and CDK5 have been considered as a major kinases that contributes to tau pathology. GSK3 consists of the highly homologous GSK3α and GSK3β, of which GSK3β was predominantly expressed in the brain (Lau et al., 1999). GSK3α is particularly abundant in the hippocampus, cerebral cortex, striatum and cerebellum, and GSK3β is expressed in almost all brain regions (Woodgett, 1990; Pandey et al., 2009). In the brains of postmortem AD and AD transgenic mouse samples, the activities of both GSK3α and GSK3β were increased, which provides a basis for GSK3 inhibitors as a therapeutic avenue for AD (Leroy et al., 2002; Terwel et al., 2008). The role of GSK3α in AD and neurodegenerative disorders was largely overlooked compared to GSK3β. The up-regulation activity of GSK3α is involved in the proteolysis processing of the Aβ peptide precursor (Phiel et al., 2003). Apart from regulating tau phosphorylation, specific inhibition of GSK3β, but not GSK3α, reduces the production of Aβ through a mechanism associated with BACE1 (Ly et al., 2013). In addition, GSK3β and CDK5 are involved in the biological process of neuron death (GO:0070997). Both GSK3β and CDK5 have been identified as core targets in the treatment of AD with UR alkaloids. Four UR alkaloids (corynoxine, RHY, isocorynoxeine and IRN) acted on GSK3β in this study, and the corynoxine showed high similarity to the selective GSK3β inhibitor AR-A014418 in molecular docking. Interestingly, a study demonstrated that RHY against MPP^+^-induced cytotoxicity by inhibiting the activity of GSK3β (Hu et al., 2018). Furthermore, treatment with IRN (20 or 40 mg/kg/day) by intragastric administration of IRN for 3 weeks significantly increased the phosphorylation of GSK3β (Ser9, inactivated form) in the hippocampus and cortex of depressed mice (Xian et al., 2019). After 3 weeks of oral gavage with IRN (20 or 40 mg/kg/day), Aβ_25-35_-induced cognitive impairment was improved in rats, and inhibition of GSK3β activity was one of the mechanisms (Xian et al., 2014). Along with GSK3β, CDK5 is considered another major tau kinase. Aβ oligomers and various neurotoxic insults increased the concentration of Ca^2+^ and activated calpain-dependent cleavage of p35 into p25 and p10. This p25 can activate and combine with CDK5 to form a stable p25/CDK5 complex, which induces various AD pathological events that are Aβ formation, tau hyperphosphorylation, synaptic plasticity, neuronal cell apoptosis (Liu L. S. et al., 2016). Furthermore, phosphorylation of CDK5 at tyrosine 15(Y15) significantly increased its kinase activity *in vivo* (Zukerberg et al., 2000). In 6-month-old APP/PS1 mice, CDK5 Y15 phosphorylation was markedly elevated in the hippocampus (Ettcheto et al., 2017). CDK5 has also been implicated in phosphorylation of APP (Thr668), making it more prone to Aβ formation (Liu et al., 2003; Liu L. S. et al., 2016). Four UR alkaloids were found to act on CDK5, including corynoxine, dihydrocorynatheine, hirsutine and isocorynoxeine. The therapeutic effect of UR against AD may be partly attributable to its effect on tau phosphorylation, in which UR alkaloids associated with GSK3β and CDK5 should be paid more attention.

NOS is divided into three subtypes, neuronal NOS (nNOS, NOS1), inducible NOS (iNOS, NOS2), and endothelial NOS (eNOS, NOS3). NOS1 is the most abundantly isoform expressed in the brain. NOS1 and NOS3 are constitutively expressed and dependent on calcium, whereas NOS2 is inducible and independent of calcium. NOS2 play an important role in neuroinflammation by generating nitric oxide (NO). In postmortem hippocampal tissue from AD brains, the transcript level of NOS2 was significantly upregulated compared with control brains (Sandusky-Beltran et al., 2021). The loss of NOS1-expressing neurons in the entorhinal cortex layer II and hippocampus of AD patients suggests that NOS1-positive neurons are susceptible to neurodegeneration (Thorns et al., 1998). Overactivation of NOS1 produces high intracellular levels of nitrite and superoxide, which react to form ROS including peroxynitrite. This oxidative stress within the cells can lead to damage to DNA, lipids, and protein modifications (Trippier et al., 2013). Overactivation of NOS1 has been implicated in neurodegeneration and therefore inhibitors of NOS1 can be used as a potential therapy.

## Conclusion

In this study, we have applied a new strategy to uncover the key UR alkaloids against AD from the perspective of pathophysiological processes. Angustoline, angustidine and isocorynoxeine were identified as the key UR alkaloids regulating Aβ production and corynoxine, isocorynoxeine, dihydrocorynatheine, IRN and hirsutine were identified as key alkaloids that regulate tau phosphorylation. The findings of this study not only contribute to a more comprehensive understanding of the key alkaloids and mechanisms of UR in the treatment of AD, but also provide candidate compounds for drug research and development for specific AD pathological processes.

## Data Availability

The original contributions presented in the study are included in the article/Supplementary Material, further inquiries can be directed to the corresponding author.

## Supplementary Material

Supplementary Figure S1 UR alkaloids that can pass through the blood-brain barrier (BBB) and enter the brain. For reference 1 (PMID: 24861762), 15 min after intravenously (i.v.) of rhynchophylline (1 or 10 mg/kg), rhynchophylline could be detected in the brain dialysate. After mice were injected intraperitoneally (i.p.) with 10 mg/kg hirsuteine and hirsutine, their distribution can be detected in the brain (PMID: 32399311). Literature 3 (PMID: 29125571) evaluated the BBB permeability of 6 UR alkaloids using an in vitro BBB model. Literature 4 (PMID: 21442303) studied the BBB permeability of 7 UR alkaloids *in vivo* and *in vitro*.

Supplementary Figure S2 Number of core targets and corresponding core targets of UR alkaloids. Twelve core targets were obtained from the PPI network according to the ranking of degrees.

Supplementary Figure S3 Interaction between GSK3B and AR-A014418 (selective GSK3β inhibitors), corynoxine by docking analysis. Hydrogen bonds between GSK3B and compounds are represented by green dashed lines. The amino acid residues of the GSK3B interacting with AR-A014418, corynoxine are shown as brown sticks and labeled in green. **(C)** The structure of the binding model shows the interaction of corynoxine with GSK3B residues in the binding pocket.

## Abbreviations

AD, Alzheimer’s disease; UR, Uncaria rhynchophylla; Aβ, Amyloid-β; BBB, Blood-brain barrier; BH, Benjamini-Hochberg; GO, Gene ontology; CFG, Convergent functional genomic; GEO, Gene Expression Omnibus; GSK3β, Glycogen synthase kinase 3β; Hacc, Hydrogen bond acceptors; Hdon, Hydrogen bond donors; HPLC, High-performance liquid chromatography; IRN, Isorhynchophylline; KEGG, Kyoto Encyclopedia of Genes and Genomes; LogP, Lipid-water partition coefficient; MW, Molecule weight; NMDA, N-methyl-D-aspartic acid; PDB, Protein Data Bank; PPI, Protein-protein interaction; Rbon, Rotatable bonds; RO5, Lipinski’s rule of five; RHY, Rhynchophylline; SD, Standard deviation.
