# Supplementary figures and images for: Therapeutic Mechanism and Key Alkaloids of Uncaria rhynchophylla in Alzheimer’s Disease From the Perspective of Pathophysiological Processes
Source: Front Pharmacol. 2021 Dec 15;12:806984. doi: 10.3389/fphar.2021.806984 (PMC8715940; doi:10.3389/fphar.2021.806984)

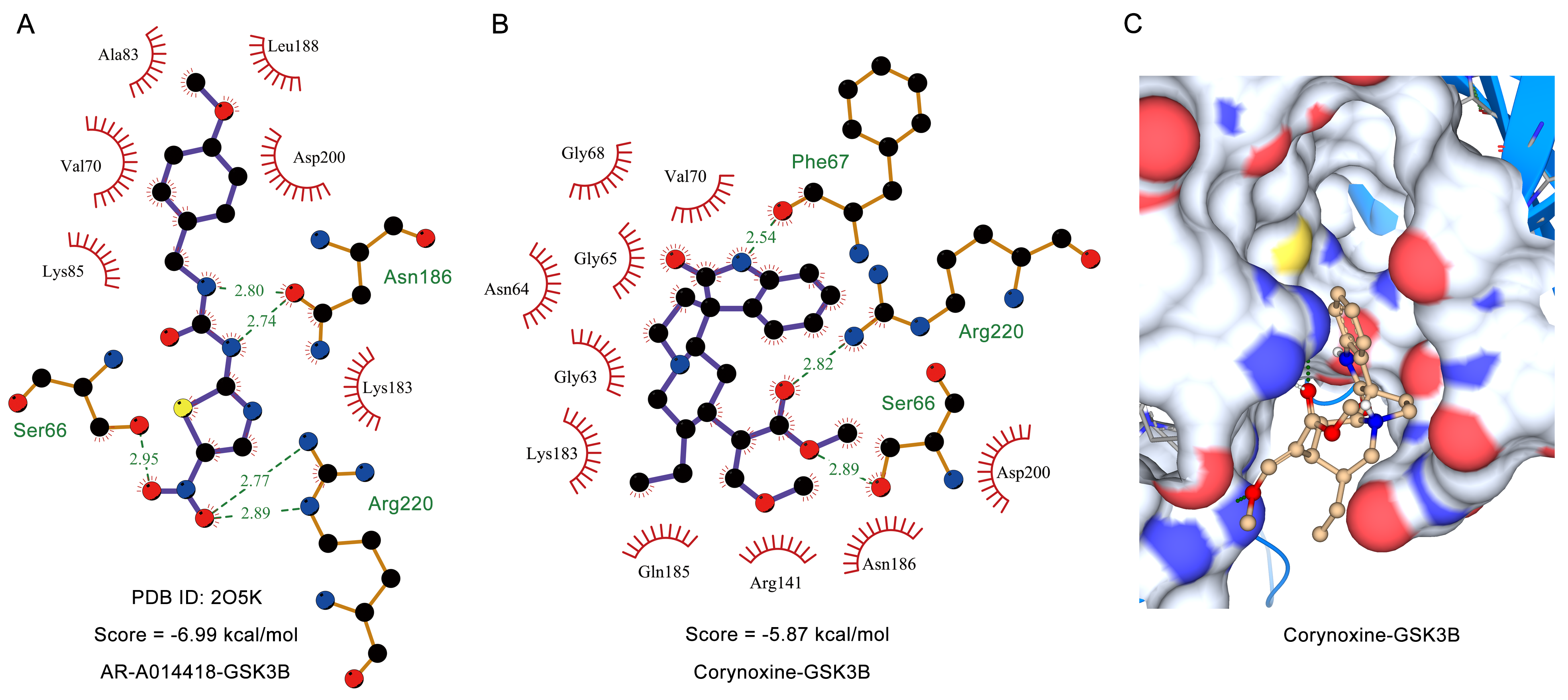

Supplement: Supplementary file 1 [file Image3.tif]

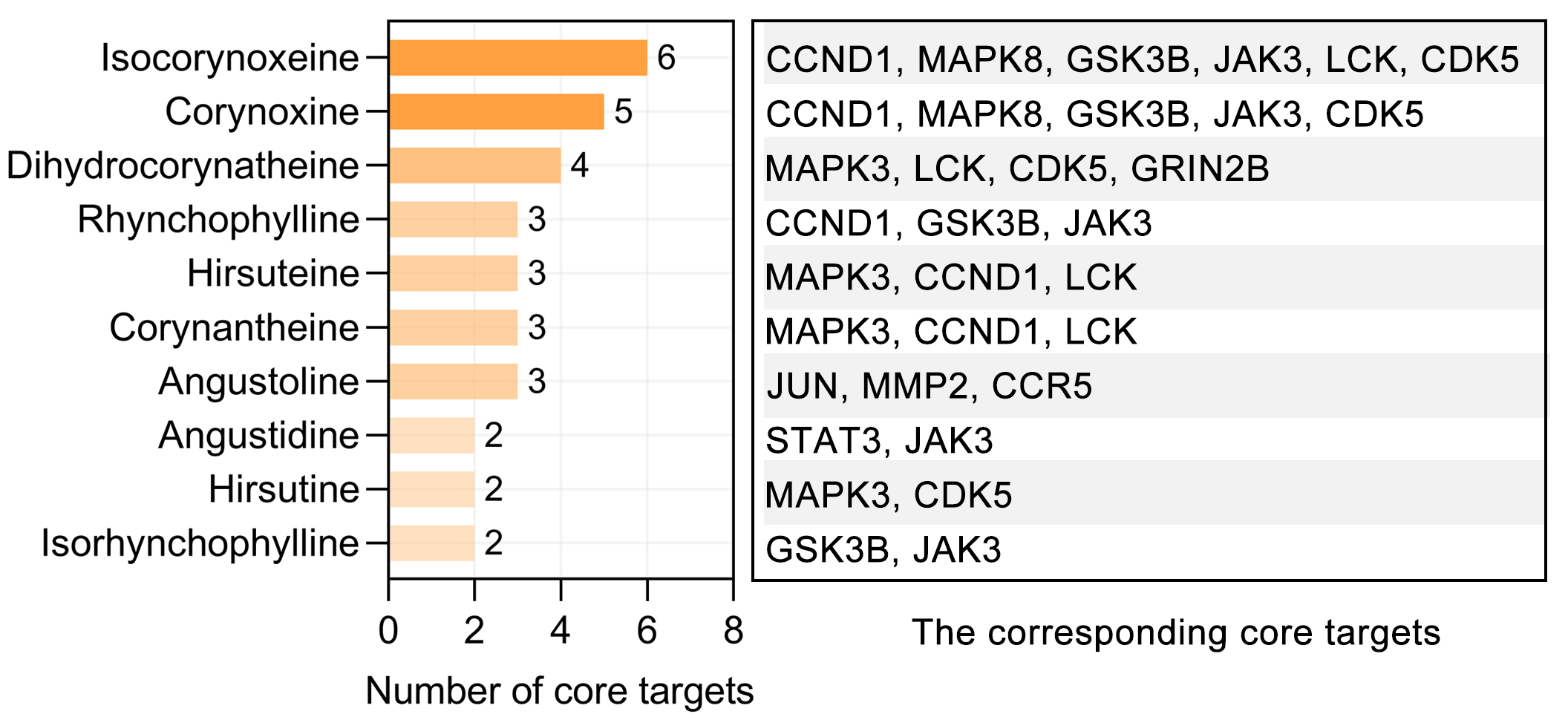

Supplement: Supplementary file 2 [file Image2.tif]

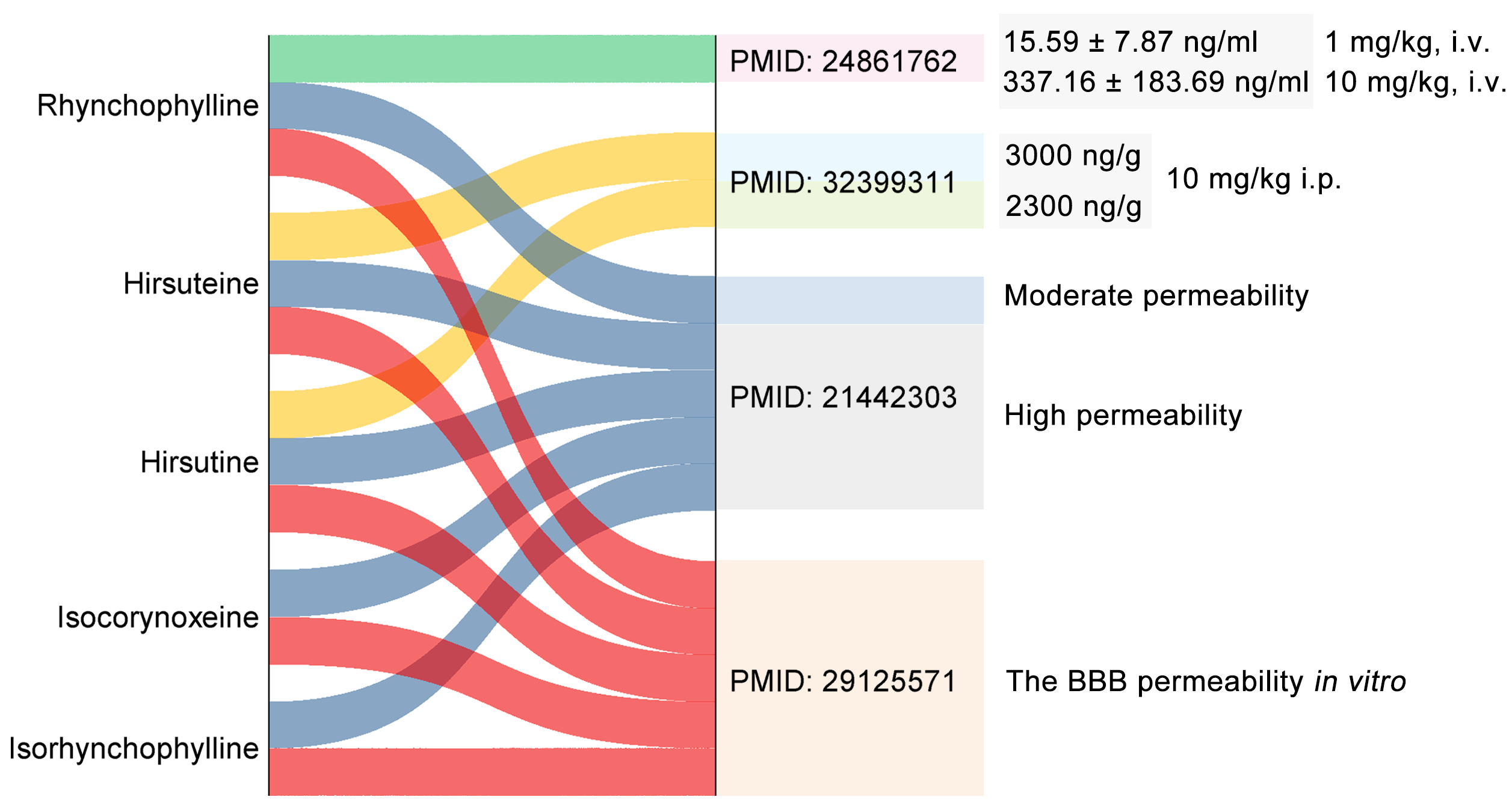

Supplement: Supplementary file 3 [file Image1.tif]
